# Hypoxia at 3D organoid establishment selects essential subclones within heterogenous pancreatic cancer

**DOI:** 10.3389/fcell.2024.1327772

**Published:** 2024-02-05

**Authors:** Koichiro Kumano, Hiromitsu Nakahashi, Pakavarin Louphrasitthiphol, Yukihito Kuroda, Yoshihiro Miyazaki, Osamu Shimomura, Shinji Hashimoto, Yoshimasa Akashi, Bryan J. Mathis, Jaejeong Kim, Yohei Owada, Colin R. Goding, Tatsuya Oda

**Affiliations:** ^1^ Department of Gastrointestinal and Hepato-Biliary-Pancreatic Surgery, Faculty of Medicine, University of Tsukuba, Tsukuba, Japan; ^2^ Ludwig Institute for Cancer Research, Nuffield Department of Medicine, University of Oxford, Oxford, United Kingdom; ^3^ International Medical Center, University of Tsukuba Hospital, Tsukuba, Japan

**Keywords:** 3D organoid, pancreatic cancer, hypoxia, tumor heterogeneity, selection bias

## Abstract

Pancreatic ductal adenocarcinoma (PDAC) is especially hypoxic and composed of heterogeneous cell populations containing hypoxia-adapted cells. Hypoxia as a microenvironment of PDAC is known to cause epithelial-mesenchymal transition (EMT) and resistance to therapy. Therefore, cells adapted to hypoxia possess malignant traits that should be targeted for therapy. However, current 3D organoid culture systems are usually cultured under normoxia, losing hypoxia-adapted cells due to selectivity bias at the time of organoid establishment. To overcome any potential selection bias, we focused on oxygen concentration during the establishment of 3D organoids. We subjected identical PDAC surgical samples to normoxia (O2 20%) or hypoxia (O2 1%), yielding glandular and solid organoid morphology, respectively. Pancreatic cancer organoids established under hypoxia displayed higher expression of EMT-related proteins, a Moffitt basal-like subtype transcriptome, and higher 5-FU resistance in contrast to organoids established under normoxia. We suggest that hypoxia during organoid establishment efficiently selects for hypoxia-adapted cells possibly responsible for PDAC malignant traits, facilitating a fundamental source for elucidating and developing new treatment strategies against PDAC.

## Introduction

Hypoxia is a key microenvironment associated with malignant traits (Schito and Semenza, 2016; Shah et al., 2020; Tao et al., 2021), including epithelial-mesenchymal transition (EMT) and chemotherapy resistance (Chang et al., 2011; Chen et al., 2016; Geyer et al., 2023). Pancreatic ductal adenocarcinoma (PDAC) is known to be extremely hypoxic due to hypovascularity and abundant stroma composed of fibroblasts and inflammatory cells (Jing et al., 2019; Schwörer et al., 2023). This severe hypoxia accelerates malignancy while typical cellular heterogeneity within tumors increases hypoxia adaptation (Mujcic et al., 2013; Rouschop et al., 2013). Thus, any hypoxia-adapted cell models for testing PDAC therapeutic strategies should be selectively extracted and cultured from clinical specimens but there is no currently reliable method.

Recently, 3D organoid culture has been widely used to simulate various organ cancers (Driehuis et al., 2019; Tuveson and Clevers, 2019; Toshimitsu et al., 2022), featuring precise control with respect to cellular selection through diverse culture conditions. However, in PDAC organoid culture, the niche dependency (Wnt, R-spondin, TGFβ) has been reported to change by cancer progression process and subtype (basal-like, classical) (Shroyer, 2016; Seino et al., 2018; Raghavan et al., 2021), suggesting the possibility of cell selection within the culture conditions. Shroyer et al. also noted that the tumor organoid system has a selection bias which is caused by competition among different clones (Shroyer, 2016). Furthermore, single cell analysis techniques have revealed more detailed heterogeneity and pointed to possible selection bias associated with culture conditions in current 3D organoid culture systems (Juiz et al., 2020; Krieger et al., 2021; Williams et al., 2023). Although intratumoral oxygen concentrations are estimated to be about 0.7% (Koong et al., 2000; McKeown, 2014), most PDAC 3D organoids are established under normoxia (O2 20%). Geyer et al. cultured PDAC organoids under hypoxia (O2 1%) and normoxia (O2 20%), and analyzed their association with therapeutic resistance (Geyer et al., 2023). To date, there are no reports of PDAC organoids established under hypoxia as a method of extraction from heterogeneous primary tumors. Following these facts, we hypothesized that a 20% oxygen culture environment within a PDAC organoid is unsuitable, introducing exclusion bias against hypoxia-adapted cells, and this selective bias would result in organoid-derived cell populations deficient in the malignant traits (e.g., chemotherapy resistance) of PDAC.

We evaluated whether the application of normoxia or hypoxia to organoids established from a single clinical specimen selectively produces a variety of tumor cell types; we thus termed pancreatic cancer organoids (PCO) established under normoxia as NORMO-PCO and those established under hypoxia as HYPO-PCO. Organoids were successfully established in both oxygen concentrations and hypoxia during organoid establishment could have the potential to select for more malignant subclones within heterogenous pancreatic cancers.

## Materials and methods

### Patient

The patient was a 54-year-old man, without genetic background, who was diagnosed with a common type of pancreatic ductal adenocarcinoma and underwent distal pancreatectomy without neo-adjuvant chemotherapy. Histopathologically, the patient was diagnosed with moderate-to-poorly-differentiated adenocarcinoma (pT2N2M0, pStageIII).

### 3D organoid culture and treatments

3D organoids were established from clinical specimens. Specimens were surgically resected from patients at University of Tsukuba Hospital with informed consent after approval by the Institutional Review Board of the University of Tsukuba Hospital (registration number: H28-090). Specimens were washed vigorously and minced into 10 mm^3^-size fragments. The fragments were digested with Liberase TH (#5401151001 Roche life science) at 37°C for 30 min and TrypLE Express Enzyme (#12605020 Thermo Fisher Scientific) at 37°C for 20 min. Approximately 2.0 × 10^4^ cells seeded with 20 µL Matrigel (#356231 BD Biosciences) domes per well on a 48-well plate. The 3D organoid culture medium consisted of Advanced DMEM/F12 (#12634010 Thermo Fisher Scientific) supplemented with HEPES (#15630080 Thermo Fisher Scientific) 10mM, Glutamax (#35050061 Thermo Fisher Scientific) 1x, Penicillin/Streptomycin (#168-23191 FUJIFILM Wako) 1x, N-acetylcysteine (#A9165 Sigma-Aldrich) 1.25 mM, and gastrin (#G9145 Sigma-Aldrich) 0.01 μM. Additionally, the medium contained following niche factors: mouse recombinant EGF (#PMG8043 Thermo Fisher Scientific) 50 nM, A83-01 (#2939 Tocris) 500 nM, Y-27632 (#253-00513 FUJIFILM Wako), IGF-1 (#590904 BioLegend)100 ng/mL, FGF10 (#100-26 PeproTech) 100 ng/mL, mouse recombinant Noggin (#250-38 PeproTech) 100 ng/mL, R-spondin1-conditioned media 10% final, and Afamin/Wnt3A (#J2-002 MBL)-conditioned media 50% final. Two plates were prepared as described above, one incubated at O2 20% (normoxia) and the other at O2 1% (hypoxia). The medium for hypoxia was previously placed in a sealed container with oxygen adsorbent at least 6 h prior to use. The media were changed every 3 or 4 days. A total of three passages were performed in medium without EGF for the purpose of enriching pancreatic cancer organoids. In order to confirm results, both cultures were eventually switched to an opposite oxygen environment and cultured for a total of three passages.

### Immunohistochemistry (IHC)

Formalin-fixed, paraffin-embedded tissues were sliced into 3-μm slide sections and deparaffinized. Antigen retrieval was performed by autoclaving sections immersed in 10 mmol/L citrate-Na buffer. The tissue sections were then treated with 3% hydrogen peroxidase in methanol for 15 min to inactivate endogenous peroxidase activity. Following blocking with normal serum, the tissues were incubated with the following primary antibodies for 1 h at room temperature: anti-Vimentin mAb (#5741, Cell Signaling Technology, 1:200, RRID: AB_10695459), anti-E-cadherin mAb (#3195, Cell Signaling Technology, 1:200, RRID:AB_ 2291471), anti-GATA6 rAb (#5851, Cell Signaling Technology, 1:400, RRID:AB_ 10705521). IHC was conducted using the avidin-biotin complex (ABC) technique with a SAB-PO kit (Nichirei Bioscience) according to the manufacturer’s instructions. Tissue sections were visualized by 3,3′-diaminobenzidine (Nichirei Bioscience) and counterstained with hematoxylin. Images were captured with a BZ-X710 microscope (Keyence Co).

### Bulk RNA-seq and gene expression quantification

RNA was extracted from organoids at sub-confluence (days 5–7) in biological triplicate. Total RNA sample libraries were prepared using RNeasy Plus Mini Kit (#74034 QIAGEN) and sequence on NovaSeq6000 using S1 Reagent Kit v1.5 flow cell (200 Cycles). Reads were demultiplexed using onboard NEBNext. Raw fastq reads for each samples were stitched using UNIX, quality controlled using fastqc (v0.11.9), then adaptor-, poly-A-, and 2 colour-trimmed as paired-end reads using trim_galore (v0.6.5; running python-base v.3.6.10, cutadapt v.2.10, java v.17.0.1, fastqc v.0.11.9, and pigz v.2.4). Processed fastq results were mapped against the *Mus musculus* (UCSC/mm39) igenomes STAR index using rna-star (v2.7.10b) using two-pass mapping with quantMode enabled, allowing for soft-clipping (Nmin = 0.2) and splicing (min 20 bp). Normalization and differential gene expression analyses were performed as previously described (PMID: 31207090, 31733993, 31595650) using edgeR (v3.40.0) glmQLFTest. Single-sample geneset enrichment and GSVA analyses were performed using R package GSVA (v1.46.0) using normalized gene expression matrices as input. Heatmaps were visualized using R package heatmap (v1.0.12) while other plots were generated using ggplot2 (v3.4.0). A modified Moffitt classification (classical vs. basal-like) was applied to each sample. The PuriST score [23] for each sample was calculated to confirm the degree of basal-like subtype.

### Gene set enrichment analysis

GSEA software (v4.3.2) was used to identify differences between NORMO-PCO and HYPO-PCO pathways and to evaluate the pathways associated with hypoxia-adapted cells. We also compared the enriched gene set under both O2 concentrations (20% and 1%) to evaluate pathways independent of the oxygen environment. Gene sets with FDRq<25 and *p*-value<0.01 were listed and graphed.

### Chemo therapeutic response

The CellTiter-Glo luminescent cell viability assay was performed on each sample according to the manufacturer’s instructions (#G7570 Promega). Briefly, cells were dissociated into single cells before plating and 1,000 cells per well were seeded with 5 µL Matrigel (#356231 BD Biosciences) domes per well on a 96-well plates in triplicate. After 24 h incubation, organoids were treated with 5-FU (#068-01401 FUJIFILM Wako) and gemcitabine (#073-06631 FUJIFILM Wako) each for 5 days. Cells were then incubated for 10 min with CellTiter-Glo reagent, and luminescence was measured using a 96-well plate reader (Thermo Fisher, Varioskan LUX). Background luminescence was measured in medium without cells and subtracted from experimental values. The IC50 of the two organoids were compared to determine chemotherapy resistance.

### Cell proliferation assay

For cell proliferation rates, approximately 5.0 × 10^3^ cells were seeded with 5 µL Matrigel domes per well on a 96-well plate in a total of 15 wells (3 rows × 5 columns). Organoids were collected daily in 3 wells with CellTiter-Glo and frozen. Luminescence was measured in a plate-wise manner with a reader on day 5. Results were graphed with proliferation rates normalized to 24 h.

### 3D organoid-derived xenografts

Mice were placed in ventilated cages with free access to water and food. Cages were changed once a week. Room temperature and humidity were maintained in the range of 20–26°C and 40–60%, respectively. Animal experiments were performed in accordance with approval from the Institutional Animal Care and Use Committee and the Regulation for Animal Experiments at the University of Tsukuba. The study also conformed to the Fundamental Guidelines for Proper Conduct of Animal Experiment and Related Activities in Academic Research Institutions under the jurisdiction of the Ministry of Education, Culture, Sports, Science and Technology (Japan). A total of 2.0 × 10^4^ cells per well were suspended in Matrigel and seeded into plates; 3 wells of organoids per site were used for grafting at sub-confluence (days 5–7). The organoids were collected and washed with Cell Recovery Solution (#354253 Corning) for 30 min, suspended with Matrigel, and implanted into subcutaneous pockets and under pancreatic capsules of CB17/Icr-scid/SCID mice (female, 6–8 weeks old, CLEA Japan). Mice were sacrificed 10 weeks after tumor seeding.

### Statistical analysis

RNA expression levels and IC50 values of chemotherapeutic response graphs were analyzed using either a Student’s t-test or Mann-Whitney U test, whichever was appropriate. The data were analyzed using GraphPad Prism 6.0. The *p*-values are expressed as follows: *p < 0.05, **p < 0.01, ***p < 0.001.

## Results

### Histopathological heterogeneity of the primary tumor

**FIGURE 1:**
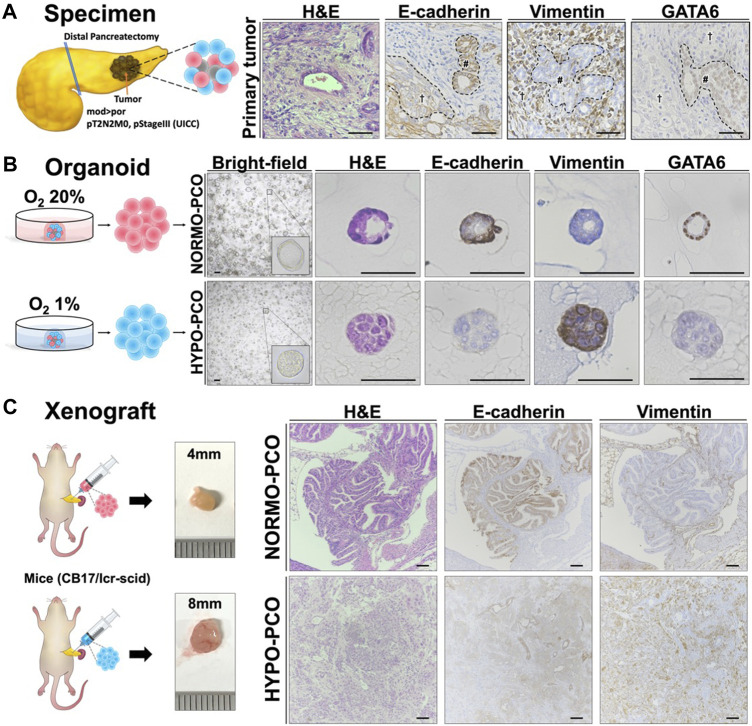
**(A)** Pancreatic cancer tissue used for organoids exhibits intratumoral heterogeneity. The histopathological diagnosis was pancreatic ductal adenocarcinoma (Pb, mod > por, pT2N2M0, pStageIII (UICC)). Immunohistochemistry staining of the primary tumor (H&E, E-cadherin, vimentin, and GATA6). Scale bars: 50 μm **(B)** Morphologic and immunohistochemical differences between NORMO-PCO and HYPO-PCO. Bright-field and immunohistochemistry staining of NORMO-PCO and HYPO-PCO (H&E, E-cadherin, vimentin, and GATA6). Scale bars: 50 μm **(C)** Proliferation and morphogenetic differences between NORMO-PCO and HYPO-PCO in pancreatic orthotopic mouse models. In both cases, tumors were identified in one of the two mice, with tumor diameters of 4 mm for NORMO-PCO and 8 mm for HYPO-PCO. No metastases were observed in both cases. Immunohistochemistry of xenograft sections from pancreatic orthotopic mouse models (H&E, E-cadherin and vimentin). Scale bars: 200 μm structured.

Organoids were generated using pancreatic cancer sections obtained from pancreatic body tail resections at our institution. Histopathologically, the primary tumor was a mixture of moderately and poorly differentiated tissues (Figure 1A). The comparatively more differentiated regions (Figure 1A, #) were immunohistochemically strongly positive for E-cadherin and GATA6, but negative for vimentin, while the poorly differentiated regions (Figure 1A, †) were weakly positive for E-cadherin and strong for vimentin but not for GATA6.

### Morphological and histological differences between NORMO-PCO and HYPO-PCO

The slice of primary tumor was mechanically and enzymatically digested and cultured in two differing oxygen concentrations: normoxia (O2 20%; NORMO-PCO) and hypoxia (O2 1%; HYPO-PCO). Both organoids were successfully established and could be cultured continuously (20+ passages). NORMO-PCO had a glandular morphology whereas HYPO-PCO had a solid morphology. Immunohistochemically, NORMO-PCO showed strong staining for E-cadherin, no staining for vimentin, and strong staining for GATA6. In contrast, HYPO-PCO showed weak staining for E-cadherin, strong staining for vimentin, and almost no staining for GATA6 (Figure 1B).

### Histopathological comparison of pancreatic orthotopic mouse models

To investigate how NORMO-PCO and HYPO-PCO behave *in vivo*, pancreatic orthotopic mouse models were created. Histopathology of these xenograft tumors showed that NORMO-PCO xenograft had adenoductal structures like typical PDAC whereas HYPO-PCO xenograft had undifferentiated regions, including some follicular structures. Immunohistochemical staining of NORMO-PCO xenograft showed strong staining for E-cadherin, none for vimentin, and strong staining for GATA6. In contrast, HYPO-PCO xenograft showed weak staining for E-cadherin, strong staining for vimentin, and almost no staining for GATA6 (Figure 1C).

### Transcriptome analysis revealed clear differences between NORMO-PCO and HYPO-PCO

**FIGURE 2:**
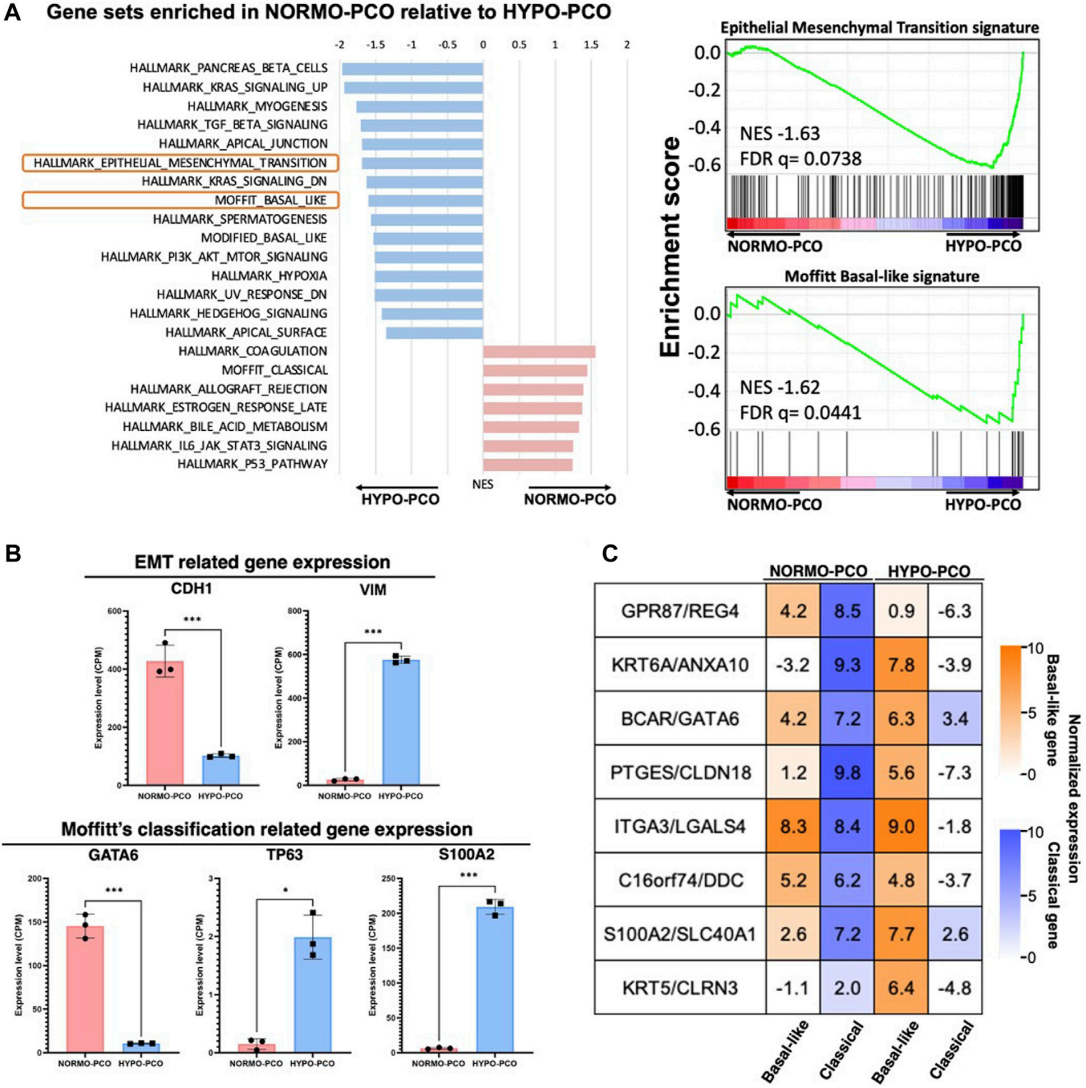
**(A)** Gene set enrichment analysis (GSEA) of NORMO-PCO relative to HYPO-PCO. EMT- and basal-like-related genes are highly expressed in HYPO-PCO. The red bar graph indicates gene sets enriched in NORMO-PCO and blue indicates gene sets enriched in HYPO-PCO. The horizontal axis shows NES value. On the right, enrichment scores for EMT (NES = 1.66, FDR = 0.073) and basal-like type (NES = 1.59, FDR = 0.065) by Moffitt classification are shown **(B)** Comparisons of expression of EMT-related genes (*CDH1*, *VIM*) and Moffit classification-related genes (*GATA6*, *TP63*, *S100A2*) in the data from RNA seq, respectively. *p < 0.05, ***p < 0.001 (unpaired Student’s t-test) **(C)** Normalized expression (log2 scale) of gene pairs assessed by purity independent subtyping of tumors (PurIST). Normalized expression (log2 scale).

Gene expression was next analyzed using next-generation RNA sequencing. Gene set enrichment analysis (GSEA) showed significant expression differences between NORMO-PCO and HYPO-PCO. Coagulation, late estrogen response, IL-6, JAK/STAT and Moffitt classical signaling were highly expressed in NORMO-PCO while pancreatic beta cells, TGF?, apical junction, epithelial-mesenchymal transition, and Moffitt basal-like signaling were highly expressed in HYPO-PCO (Figure 2A). The expression of *CDH1*, *VIM*, *GATA6*, *S100A2*, and *TP63* as representative genes of EMT and basal-like type (Moffitt et al., 2015) were compared. We found that expression of *VIM*, *S100A2*, and *TP63* was significantly higher (*VIM*, *S100A2*: *p* < 0.001, *TP63*: *p* < 0.05) while *CDH1* and *GATA6* expression was significantly lower (*CDH1*, *GATA6*: *p* < 0.001) in HYPO-PCO *versus* NORMO-PCO (Figure 2B). Purity independent subtyping of tumor (PurIST) scoring (Rashid et al., 2020), from the ratios of eight classifier gene pairs, showed NORMO-PCO was a classical subtype while HYPO-PCO was a basal-like subtype (Figure 2C).

### Evaluation of response to chemotherapy in NORMO-PCO and HYPO-PCO *in vitro*

**FIGURE3:**
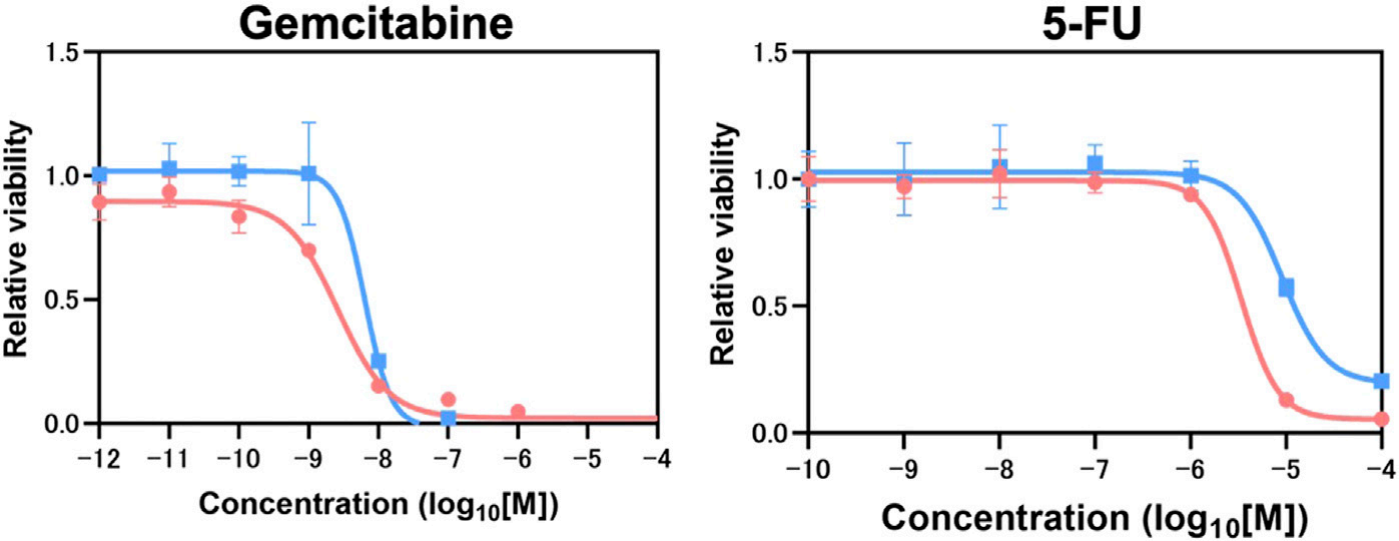
Chemotherapy resistance experiments which conducted in biological triplicate revealed 5-FU resistance in HYPO-PCO. Cell viability was analyzed by using CellTiter-Glo 2.0. Dose response curves for gemcitabine range from 1.0 × 10^−12^ to 1.0 × 10^−4^ mol/L and 5-FU range from 1.0 × 10^−10^ to 1.0 × 10^−4^. Red dots indicate NORMO-PCO; blue dots indicate HYPO-PCO. Drug concentration is in log_10_ [M].

Next, molecular response to chemotherapy (gemcitabine and 5-FU) were evaluated. There were no significant differences in responses to gemcitabine treatment (NORMO-PCO IC50 = 10^−8.6;^ HYPO-PCO IC50 = 10^−8.18^) but significant differences in 5-FU responses were observed (NORMO-PCO IC50 = 10^−5.87^; HYPO-PCO IC50 = 10^−5.15^ [*p* < 0.05]) (Figure 3B; Supplementary Figure S1).

### Switching oxygen concentrations indicates that NORMO-PCO and HYPO-PCO are genotypically distinct

**FIGURE 4:**
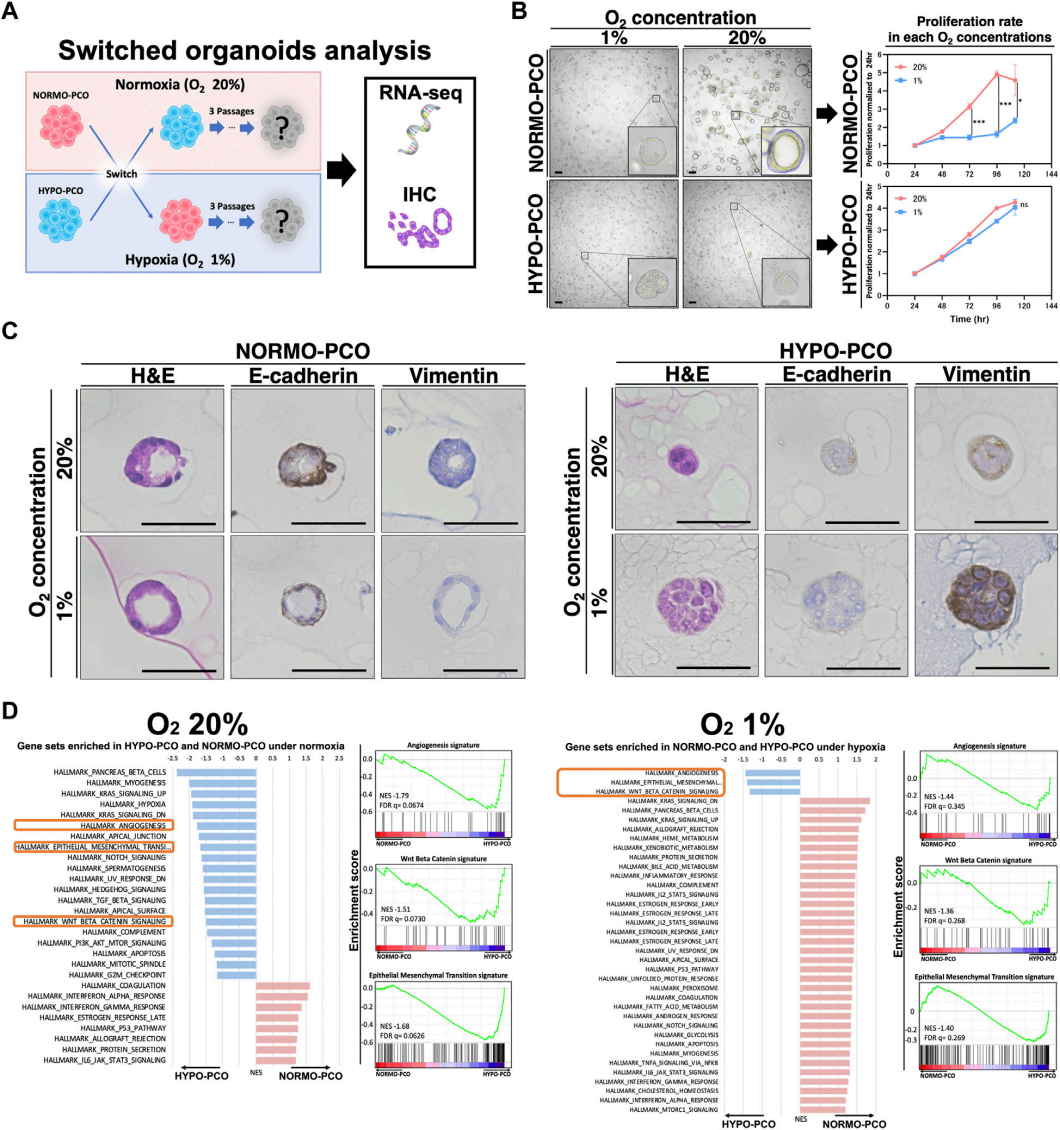
**(A)** Analysis after switching oxygen conditions reveals that NORMO-PCO and HYPO-PCO are irreversible. Experimental scheme. NORMO-PCO was cultured in hypoxia and HYPO-PCO in normoxia for three passages before morphology and transcriptome analysis **(B)** Bright field microscopy under each oxygen concentration. Scale bars: 50 μm. The growth curves of the organoids under each oxygen concentration. Proliferation normalized to 24 h **(C)** Immunohistochemistry of NORMO-PCO and HYPO-PCO (H&E, E-cadherin, vimentin). Scale bars: 50 μm **(D)** Comparison of GSEA analysis of NORMO-PCO and HYPO-PCO in matching oxygen niches. The red bar indicates gene sets enriched in NORMO-PCO, and blue indicates gene sets enriched in HYPO-PCO.

Considering the possibility that the difference between these two organoids is affected by oxygen concentration, the oxygen concentrations of each organoid were switched before immunohistochemical and transcriptomic analyses to examine adaptation responses (Figure 4A). When NORMO-PCO was incubated in 1% oxygen, the proliferation rate was significantly slower than those incubated in 20% oxygen, but no morphological changes were observed. Similarly, when HYPO-PCO was incubated normoxically, the proliferation rate was not significantly changed and morphology remained unchanged (Figure 4B). There was no significant change in the size of either organoid under different oxygen concentrations (Supplementary Figure S2). Immunohistochemistry for EMT markers (E-cadherin, VIM) were performed (Figure 4C). Hypoxic NORMO-PCO showed a slight decrease in E-cadherin expression while vimentin expression increased but remained significantly lower than HYPO-PCO. The expression of EMT-related genes in NORMO-PCO was affected by but not equivalent to HYPO-PCO after the switch. In contrast, when HYPO-PCO was incubated normoxically, E-cadherin expression remained unchanged and vimentin expression decreased but remained significantly higher than NORMO-PCO.

GSEA results of NORMO-PCO and HYPO-PCO after switching were compared. EMT, angiogenesis, and β-catenin signatures were significantly enriched in HYPO-PCO in both oxygen concentrations (Figure 4D) while many hypoxia-related genes (ex. *GLUT1*, *GLUT3*, *NDRG1* and *CA9*) were similar in both NORMO-PCO and HYPO-PCO (Supplementary Figure S3).

## Discussion

By applying normoxia and hypoxia selective culture to 3D organoids, we successfully obtained two different clones regarding morphology, gene expression, and drug resistance. HYPO-PCO clones had a solid morphology, basal-like type characteristics, higher expression of EMT-related genes, and enhanced 5-FU resistance in contrast to the NORMO-PCO clones (cystic, 5-FU sensitive, classical type).

The morphology of the primary PDAC tissue we used for 3D organoid establishment was quite heterogenous in differentiation. Such intratumor heterogeneity is well-reported with highly differentiated tissues exhibiting epithelial characteristics and poorly differentiated tissues exhibiting mesenchymal characteristics (Hayashi et al., 2020; Milan et al., 2021). The *in vitro* morphology of our two 3D organoids demonstrated that NORMO-PCO had cystic morphology, E-cadherin (+), vimentin (−), and was similar to highly differentiated regions of original clinical PDAC tissue, while HYPO-PCO had solid morphology, E-cadherin (±), vimentin (+), and was similar to poorly differentiated regions. We thus assumed that NORMO-PCO was selectively amplified from highly differentiated regions and HYPO-PCO was equally selected from poorly differentiated regions. This notion was further supported by *in vivo* pancreatic orthotopic mouse models that revealed NORMO-PCO histology as similar to well-differentiated regions of the original tumor while HYPO-PCO showed similar histology to the poorly differentiated regions of the original tumor. This selective effect demonstrates that cells within highly differentiated regions prefer normoxia while cells from poorly differentiated regions prefer hypoxia and possess unique molecular profiles to withstand and thrive within such a rigorous microenvironment.

RNA-seq was performed to understand the differences in gene expression between these morphologically distinct NORMO-PCO and HYPO-PCO cells. As expected, gene expression clearly differed, with the hallmark gene sets enriched in HYPO-PCO as K-RAS signaling, TGF-β signaling, epithelial-mesenchymal-transition, Hedgehog, and basal-like subtype. In contrast, NORMO-PCO had high expression of immune system-related genes (such as IL-6, allograft, and coagulation) with classical characteristics. These results indicate that NORMO-PCO and HYPO-PCO had unique molecular signatures reflective of their respective oxygenation conditions.

In 2015, Moffitt et al. (2015) proposed that pancreatic cancer gene expression diverged into two subtypes, classical and basal-like; later, a degree of GATA6 expression was reported to distinguish these subtypes (de Andrés et al., 2023; O'Kane et al., 2020). Similarly, our organoids clearly follow this story. In fact, NORMO-PCO (classical subtype) exhibited high expression of GATA6 while HYPO-PCO (basal-like subtype) exhibited low expression by immunohistochemical staining and RNA-seq. Recent reports from single-cell analyses of pancreatic cancer nodules clearly revealed intratumor heterogeneity where classical subtype cells and basal-like subtype cells coexist within the same tumor (Juiz et al., 2020; Krieger et al., 2021; Williams et al., 2023). Furthermore, it was revealed that hypoxia-related genes are enriched in the basal-like subtype, indicating that these cells may thrive in hypoxic regions (Abou Khouzam et al., 2021). This subtype and associated gene expression are thus likely to be responsible for malignant traits due to their derivation from a more aggressive population within the heterogeneous primary PDAC.

To estimate the malignant potential of these two 3D organoids, chemotherapeutic response was compared. There was a significant change in responsiveness to 5-FU between NORMO-PCO and HYPO-PCO, indicating that HYPO-PCO is more resistant to 5-FU chemotherapy. Our data are supported by a comparative chemotherapy resistance study that revealed the basal-like subtype to be more 5-FU resistant than the classical subtype (O'Kane et al., 2020; Martinelli et al., 2017). These results highlight differences in heterogenous chemosensitivity *in vivo* and testing both NORMO-PCO and HYPO-PCO will be meaningful for finding eradicative drug combinations for both populations in upcoming tailored drug screenings.

Next, we evaluated whether morphology and gene expression were reversible *via* switching oxygen concentrations, revealing that NORMO-PCO maintained its distinct morphology, gene expression, and classical subtype traits in hypoxia. Similarly, HYPO-PCO maintained its native traits even under normoxia, with irreversible expression of EMT, angiogenesis, and Wnt/β-catenin-related genes. The only exception was the expression of hypoxia-related genes (e.g., *GLUT1*, *GLUT3*, *NDRG1* and *CA9*) (Angst et al., 2006; Balamurugan, 2016; Logsdon et al., 2016) that had significantly higher expression when NORMO-PCO and HYPO-PCO were cultured in hypoxia *versus* normoxia. The results indicate that NORMO-PCO and HYPO-PCO were independent and different clonal populations that are not affected solely by oxygen. Furthermore, the EMT, angiogenesis, and β catenin-related gene expression mediated by our hypoxic culture condition were found to be important and durable markers of hypoxia-adapted cells. Regarding the proliferation of NORMO-PCO and HYPO-PCO at each oxygen concentration, the proliferation rate of NORMO-PCO in 1% oxygen was significantly slower than in 20% oxygen whereas the proliferation rate of HYPO-PCO in 20% oxygen was not significantly changed. A possible reason why the proliferation rates did not correlate with the size of organoids is that the HYPO-PCO organoids had no lumen, plus densely packed cells, resulting in a high number of cells per unit organoid.

Recently, 3D organoid culture systems have been widely used for their ability to simulate the actual conditions of living organs and cancers. It should be noted, however, that generation efficiency of organoids by establishment in normoxia (20% O2) is not reflective of the hypoxic environment in which pancreatic cancer exists in patients. As a result, the inherent selective bias of these widely used organoids may exclude hypoxic cells which should be targeted for treatment. To better reflect actual clinical conditions, analysis and targeting of the hypoxia-resistant and drug-resistant cells that are responsible for the malignant phenotype of PDAC should be a priority. While there is a limitation that epigenetic changes induced by oxygen conditions at the time of establishment may be maintained thereafter, in any case, establishing organoids under hypoxia, in addition to normoxia, may more accurately reproduce the known heterogeneity of PDAC tumors and reveal new insights for targeting infamous PDAC malignant traits.

## Conclusion

We report success in obtaining two geno- and phenotypically distinct pancreatic cancer 3D organoid clones by simply adjusting oxygen levels at initial establishment. Hypoxia, compared to conventional normoxia, effectively selects poorly differentiated cells with Moffitt-basal like subtype characteristics that should maintain PDAC malignant traits. Thus, more accurately simulating clinical PDAC intratumor heterogeneity with both NORMO-PCO and HYPO-PCO will bring depth to researches against recalcitrant pancreatic cancer.

## Data Availability

The data presented in the study are deposited in the https://www.ncbi.nlm.nih.gov/geo/query/acc.cgi?acc=GSE240649 repository, accession number GSE240649.
