# Supplementary material for: Hypoxia at 3D organoid establishment selects essential subclones within heterogenous pancreatic cancer
Source: Front Cell Dev Biol. 2024 Feb 5;12:1327772. doi: 10.3389/fcell.2024.1327772 (PMC10875002; doi:10.3389/fcell.2024.1327772)
Supplement: Supplementary file 2 [file DataSheet1.docx]

Supplementary Material

# Supplementary Figures

## Supplementary Figures 1

**Supplementary Figure 1.** Brightfield images of organoids on day 5 after treatment with each concentration; gemcitabine range from 1.0×10-12 to 1.0×10-4 mol/L and 5-FU range from 1.0×10-10 to 1.0×10-4.

## Supplementary Figures 2


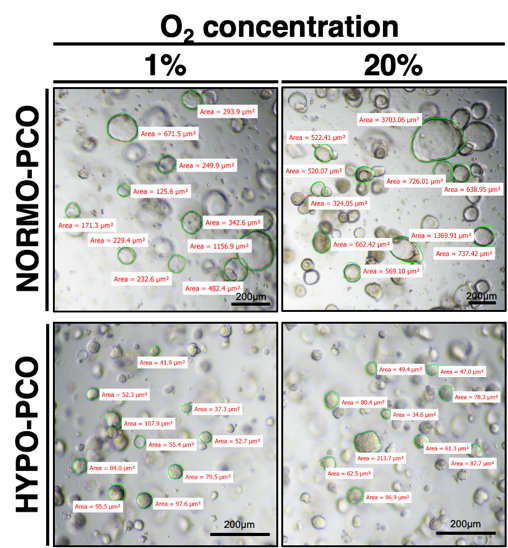

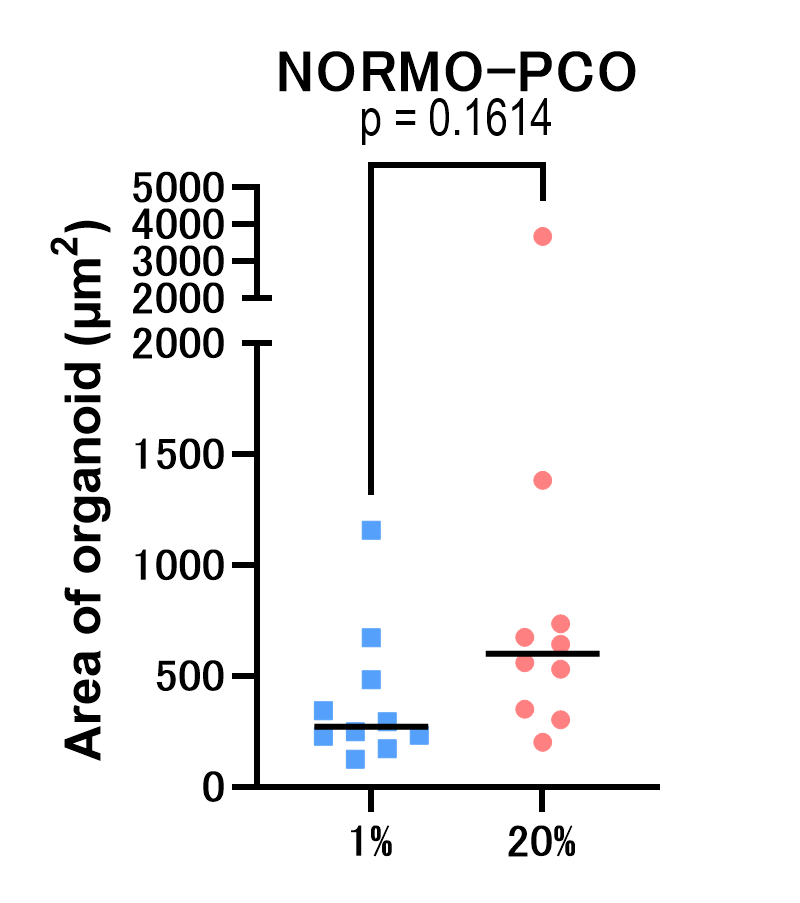

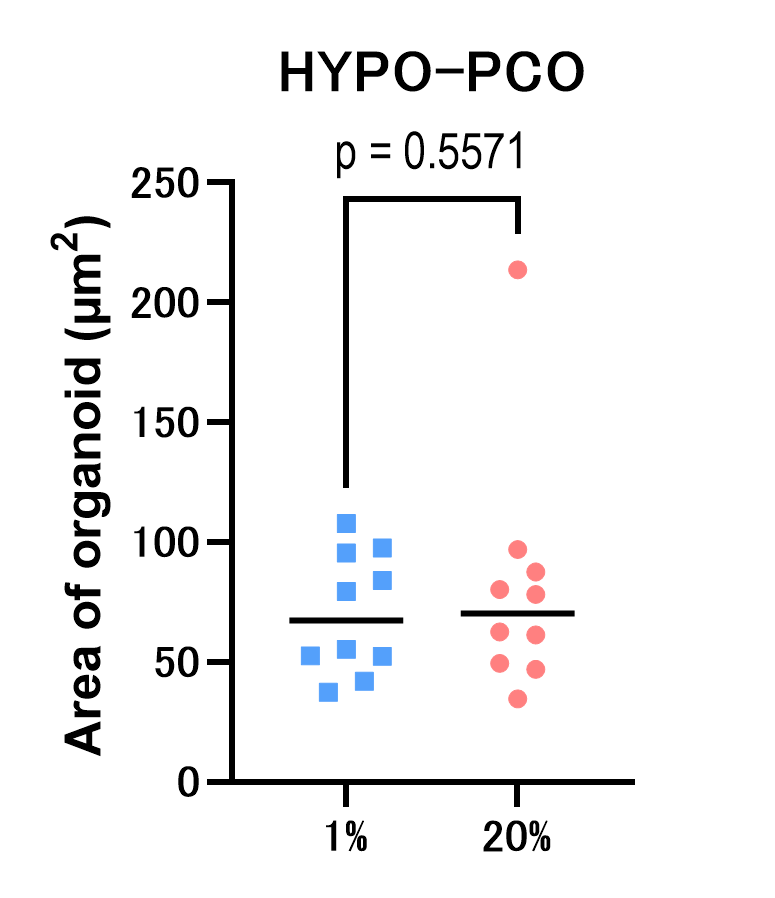


**Supplementary Figure 2.** the size of 10 randomly selected organoids on day7 by ECLIPSE Ti2-U (Nikon). There was no significant change in the size of both organoids under different oxygen concentrations (unpaired t test).

## Supplementary Figures 3

**Supplementary Figure 3.** Comparisons of expression of hypoxia-related genes (*GLUT1, GLUT3, NDRG1* and *CA9*) in NORMO-PCO and HYPO-PCO under both oxygen conditions (O2 20% and 1%). All the data were significantly changed reversibly (unpaired t test).
